# The Role of ^18^F-FDG PET/CT in the Evaluation of Peritoneal Thickening of Undetermined Origin

**DOI:** 10.1097/MD.0000000000003023

**Published:** 2016-04-18

**Authors:** Ruohua Chen, Yumei Chen, Liu Liu, Xiang Zhou, Jianjun Liu, Gang Huang

**Affiliations:** From the Department of Nuclear Medicine, Ren Ji Hospital, School of Medicine, Shanghai Jiao Tong University (RHC, YMC, LL, XZ, JJL, GH); and Department of Cancer Metabolism, Institute of Health Sciences, Chinese Academy of Sciences and Shanghai Jiao Tong University School Medicine, Shanghai, China (GH); Shanghai University of Medicine & Health Sciences (SHMHS) (GH).

## Abstract

The aim of this study was to assess the value of ^18^F-fluorodeoxyglucose (FDG) positron emission tomography (PET)/computed tomography (CT) for the differentiation of peritoneal thickening of undetermined origin.

This retrospective study included 103 patients (44 men and 59 women, age 59.2 ± 14.8 years) who had undergone ^18^F-FDG PET/CT for the evaluation of peritoneal thickening of undetermined origin. All ^18^F-FDG PET/CT images were visually interpreted, and the maximal standardized uptake values (SUV_max_) were measured. We compared the role of ^18^F-FDGPET/CT with that of CT alone in detecting peritoneal thickening of undetermined origin. We also compared the differences between malignant and tuberculous peritoneal thickening in PET/CT parameters and clinical characteristics.

The sensitivity, specificity, positive predictive value (PPV), negative predictive value (NPV), and accuracy in detecting the primary cause of the peritoneal thickening were 76.2%, 78.9%, 94.1%, 42.9%, and 81.2%, respectively, for ^18^F-FDG PET/CT, and 58.3%, 84.2%, 94.2%, 31.4%, and 63.1%, respectively, for CT imaging. Malignant peritoneal thickening had significantly higher SUV_max_ than nontuberculous benign peritoneal thickening. However, tuberculous peritoneal thickening also had a high SUV_max_. There were some factors that were significantly different between patients with tuberculous peritoneal thickening and those with malignant peritoneal thickening in our study; these included age, pattern of peritoneal thickening, and presence of ascites.

^18^F-FDG PET/CT is useful for detecting the underlying cause of peritoneal thickening. Special attention should be paid to peritoneal tuberculosis, which has a high SUV_max_ and may mimic malignant peritoneal thickening. Multiple PET/CT parameters which were different in patients with tuberculous and malignant causes could be taken into consideration to make the differential diagnosis.

## INTRODUCTION

Peritoneal thickening is a persistent problem that can be caused by several diseases.^1–4^ Identification of the cause of peritoneal thickening is often critical for optimal management and prognostication. Serum and peritoneal fluid biochemical tests and fluid cytology have low positive rates. The utility of peritoneal biopsy is limited because of its invasiveness, although it has high diagnostic accuracy.^5^ Conventional imaging methods used to characterize peritoneal thickening, such as unenhanced computed tomography (CT) attenuation, enhanced CT, or magnetic resonance imaging, have some limitations in differentiating between the presence and absence of primary lesions, even if peritoneal thickening is identified.^6,7^

^18^F-fluorodeoxyglucose (FDG) positron emission tomography (PET) has been widely used to differentiate between benign lesions and malignant tumors.^8–11^ Integrated PET/CT is not only complementary to conventional imaging, but also may be more sensitive because the metabolic alterations of malignant tumors may precede gross anatomical changes.^8,12^ However, ^18^F-FDG accumulates not only in malignant tumors but also in several benign lesions that may mimic malignant lesions and thus limit the specificity of ^18^F-FDG PET/CT imaging.^13^ So far, few studies have examined the role of ^18^F-FDG PET/CT in the evaluation of peritoneal thickening of undetermined origin. The purpose of this study was to assess the value of ^18^F-FDG PET/CT in determining the cause of peritoneal thickening.

## MATERIALS AND METHODS

### Patients

We performed a retrospective analysis of ^18^F-FDG PET/CT obtained in 103 patients (44 men and 59 women; age range, 23–77 years; mean age, 59.2 ± 14.8 years) with peritoneal thickening. The examinations were performed between January 2010 and April 2015 to determine the primary cause of the peritoneal thickening and to differentiate malignant from benign peritoneal thickening. The Institutional Review Board of Shanghai Jiao Tong University–affiliated Ren Ji Hospital approved this study, and all patients gave written informed consent.

There were 84 patients with malignant peritoneal thickening and 19 patients with benign peritoneal thickening among the 103 patients. Among the 84 patients with malignant peritoneal thickening, the presence of primary malignant lesions was diagnosed by pathologic examination and clinical follow-up over 6 months. Among the 19 patients with benign peritoneal thickening, pathologic examinations confirmed the cause in 7 patients. In the other 12 patients, the causes of the peritoneal thickening included peritoneal tuberculosis (7), bacterial peritonitis (2), nephritic syndromes (2), and hepatic cirrhosis (1); these cases were diagnosed by clinical follow-up over 6 months.

### PET/CT

Whole body scanning was performed by using a whole body PET/CT scanner (Biograph mCT; Siemens). All patients received an intravenous injection of 3.7 MBq/kg of ^18^F-FDG after fasting at least 6 hours and resting for 1 hour. The mean uptake time was 50 ± 6 minutes. Blood glucose measurements were obtained in all patients before the administration of ^18^F-FDG and were less than140 mg/dL at the time of injection.

CT was performed on the 64-slice CT (Biograph mCT; Siemens) without contrast administration. A standardized protocol was followed, involving120 kV, 140 mA, and a section thickness of 5.0 mm, which was matched to the section thickness of the PET images. PET image datasets were reconstructed iteratively with CT data for attenuation correction.

### Image Interpretation

PET/CT images were assessed by 2 experienced nuclear medicine physicians on a workstation (Medx) in all standard planes. They had at least 5 years experiences in PET/CT images. Where discrepancies occurred, they reached a consensus. When increased uptake, greater than the background activity of the organ, was identified, the abnormal focal lesion was considered to be a potential primary lesion combined with the patients’ age, sex, past history, gastroscopy, and enteroscopy. They were blinded to the gold standard outcome. The gold standard outcome was obtained by biopsy or clinical follow-up. For quantitative analysis, irregular regions of interest were placed over the most intense area of ^18^F-FDG accumulation. The maximal standardized uptake value (SUV_max_) was calculated using the following formula: maximum pixel value with the decay-corrected region-of-interest activity (MBq/mL)/(injected dose [MBq]/body weight [g]). The CT manifestations of the primary origin were evaluated according to the standard CT diagnostic routine. We then assessed the diagnostic accuracy of ^18^F-FDG PET/CT versus CT alone in the detection of original lesions. The diagnostic endpoints included: whether PET/CT or CT can detect peritoneal thickening; whether PET/CT can identify the site of primary for malignant peritoneal thickening; and whether the SUV_max_ values can distinguish malignant from benign peritoneal thickening or differentiate malignant from nontuberculous benign thickening.

### Statistical Analysis

The primary analysis was of the diagnostic performance of ^18^F-FDG PET/CT and of CT imaging. The performance was assessed by calculating the sensitivity, specificity, positive predictive value (PPV), negative predictive value (NPV), and accuracy. The sensitivity was defined as the number of true-positive decisions of malignant primary lesions diagnosed by PET/CT or CT divided by the number of actually malignant primary lesions. The specificity was defined as the number of true-negative decisions of no malignant primary lesions diagnosed by PET/CT or CT divided by the number of actually no malignant primary lesions. PPV is defined as the number of true-positive decisions of malignant primary lesions diagnosed by PET/CT or CT divided by the number of malignant primary lesions diagnosed by PET/CT or CT. NPV is defined as the number of true-negative decisions of no malignant primary lesions diagnosed by PET/CT or CT divided by the number of no malignant primary lesions diagnosed by PET/CT or CT. The data were represented as means ± standard deviation. Statistical differences between the groups were compared using one-way ANOVA and the *t*-test. *P* < 0.05 was considered statistically significant. All statistical analysis was performed using SPSS version 13.0 (SPSS Inc., Chicago, IL).

## RESULTS

### Distribution of Primary Lesions

Among the 103 patients, 84 were found to have malignant diseases: these included gastric cancer (20), ovarian cancer (12), pancreatic cancer (9), colon carcinoma (8), lymphoma (7), liver cancer (7), unknown primary lesions (7), lung cancer (5), carcinoma of the small intestine (3), gallbladder carcinoma (3), uterine cancer (2), and malignant peritoneal mesothelioma (1). The other 19 patients were found to have benign lesions, which included peritoneal tuberculosis (12), bacterial peritonitis (4), nephritic syndromes (2), and hepatic cirrhosis (1).

### Detection of Primary Lesions

**FIGURE 1:**
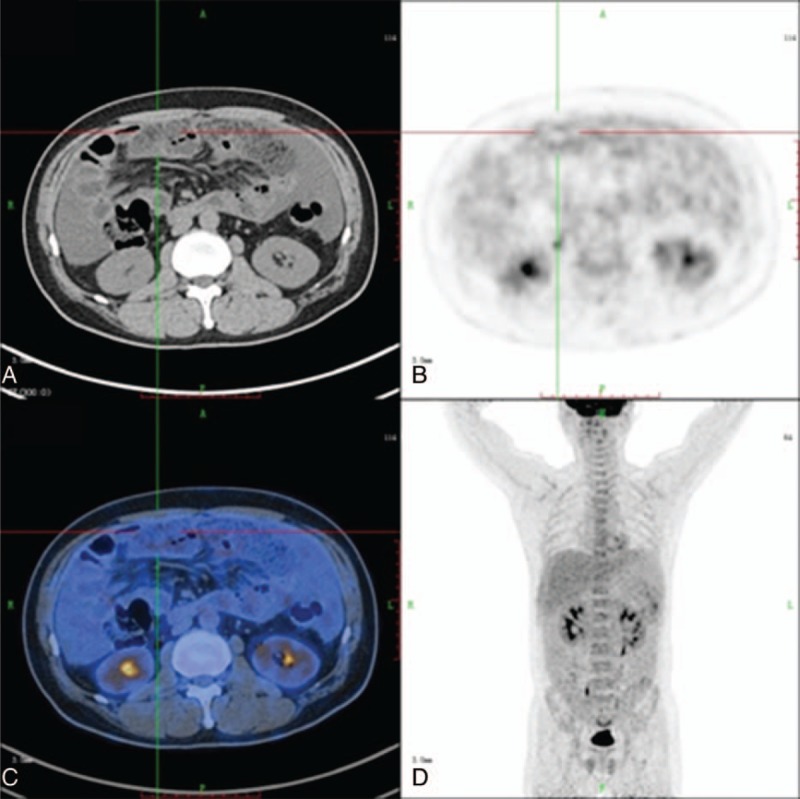
Images of 51-year-old man who presented with abdominal pain for 3 months: axial CT (A), axial PET (B), axial fused PET/CT (C), and 3D PET (D). The patient had serum CA12-5 of 355 U/mL. Noninvasive examinations could not detect the primary cause of the peritoneal thickening. PET/CT images show normal uptake in the gastric area (SUV_max_ of 1.5). However, gastroscopy was repeated, and biopsy confirmed a malignant gastric lesion. CT = computed tomography, PET = positron emission tomography, SUV_max_ = maximal standardized uptake value.

**FIGURE 2:**
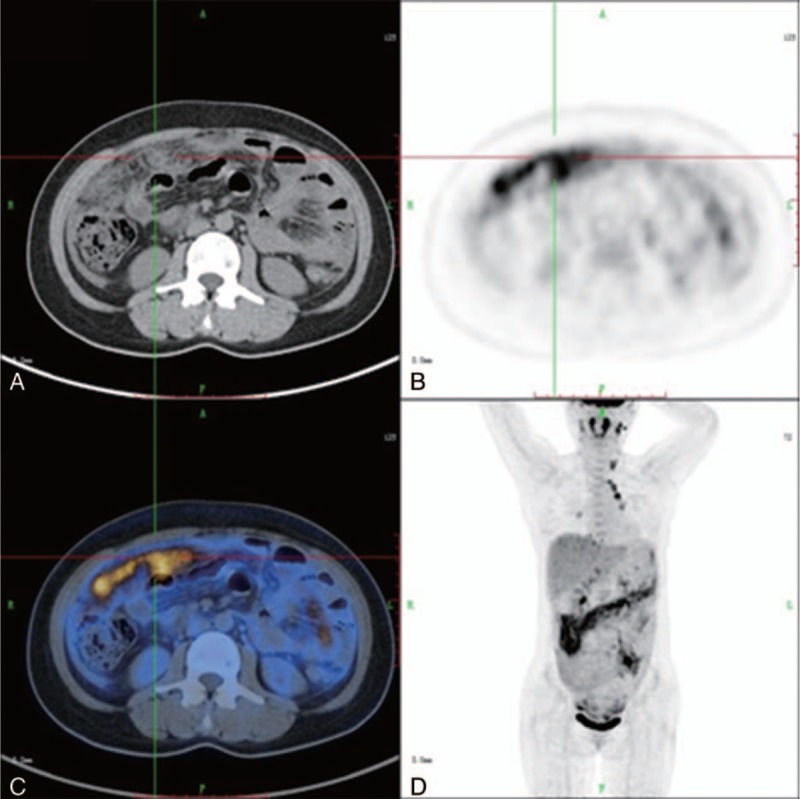
PET/CT images of a false-positive case, a 41-year-old woman who presented with peritoneal thickening: axial CT (A), axial PET (B), axial fused PET/CT (C), and 3D PET (D). The patient had abdominal pain and distention for 3 weeks. She had serum CA12-5 of 1126 U/mL, normal serum α-fetoprotein, normal carcinoembryonic antigen, and normal CA19-9. PET/CT shows increased ^18^F-FDG uptake in the peritoneum (SUV_max_ of 5.6), mimicking a malignant lesion. Peritoneal biopsy confirmed that the patient had peritoneal tuberculosis. CT = computed tomography, FDG = fluorodeoxyglucose, PET = positron emission tomography, SUV_max_ = maximal standardized uptake value.

Among the 84 patients with malignant peritoneal thickening, PET/CT detected the primary lesion in 64 (76.2%). In the 20 patients in whom PET/CT gave false-negative results, malignant disease was confirmed by pathologic examination; these patients included 7 with unknown primary lesions, 3 with poorly differentiated ovarian adenocarcinoma, 3 with carcinoma of the small intestine, 2 with gastric carcinoma (Figure 1), 2 with colon carcinoma, 2 with lung cancer, and 1 with liver cancer. There were 4 patients with false-positive results. In these 4 patients, increased ^18^F-FDG uptake in the peritoneum was initially considered to represent peritoneal metastasis from unknown primary tumors, but peritoneal biopsy confirmed peritoneal tuberculosis (Figure 2).

**FIGURE 3:**
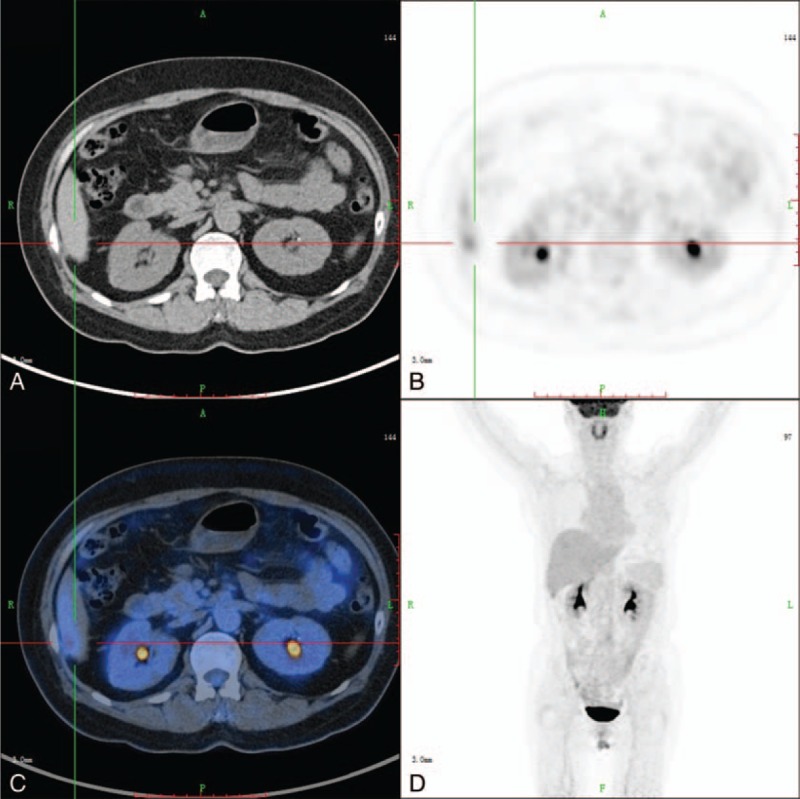
Images of 70-year-old woman who presented with abdominal pain for 6 months: axial CT (A), axial PET (B), axial fused PET/CT (C), and 3D PET (D). CT examinations could not detect the hepatic peritoneal metastasis from ovarian cancer. PET/CT images show high uptake in the hepatic peritoneum (SUV_max_ of 3.5). Metastasis in the hepatic peritoneum was finally confirmed by pathology. CT = computed tomography, PET = positron emission tomography, SUV_max_ = maximal standardized uptake value.

The sensitivity, specificity, PPV, NPV, and accuracy in detecting the primary cause of peritoneal thickening were 76.2%, 78.9%, 94.1%, 42.9%, and 81.2%, respectively, for ^18^F-FDG PET/CT, and 58.3%, 84.2%, 94.2%, 31.4%, and 63.1%, respectively, for CT imaging (Table 1). Thus, compared with conventional CT imaging, PET/CT appears to have superior sensitivity and accuracy for the detection of primary malignant lesions. Figure 3 shows the images of 1 patient with discordant results for CT and PET/CT. In this patient with ovarian cancer, CT could not confirm metastasis in the hepatic peritoneum; however, PET detected high uptake in the hepatic peritoneum, suggesting a metastatic lesion, which was finally confirmed by pathology.

**TABLE 1:**
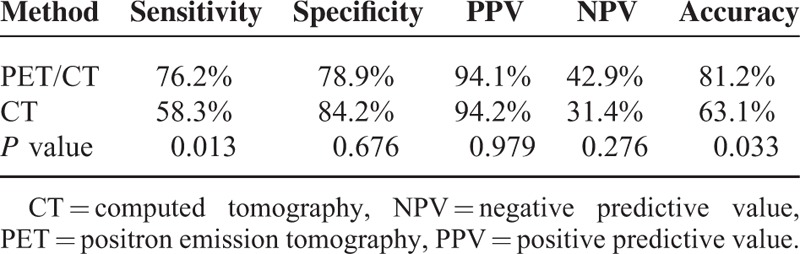
The Sensitivity, Specificity, PPV, NPV, and Accuracy in Detecting the Primary Cause of Peritoneal Thickening

### Characteristics of Peritoneal Thickening

**FIGURE 4:**
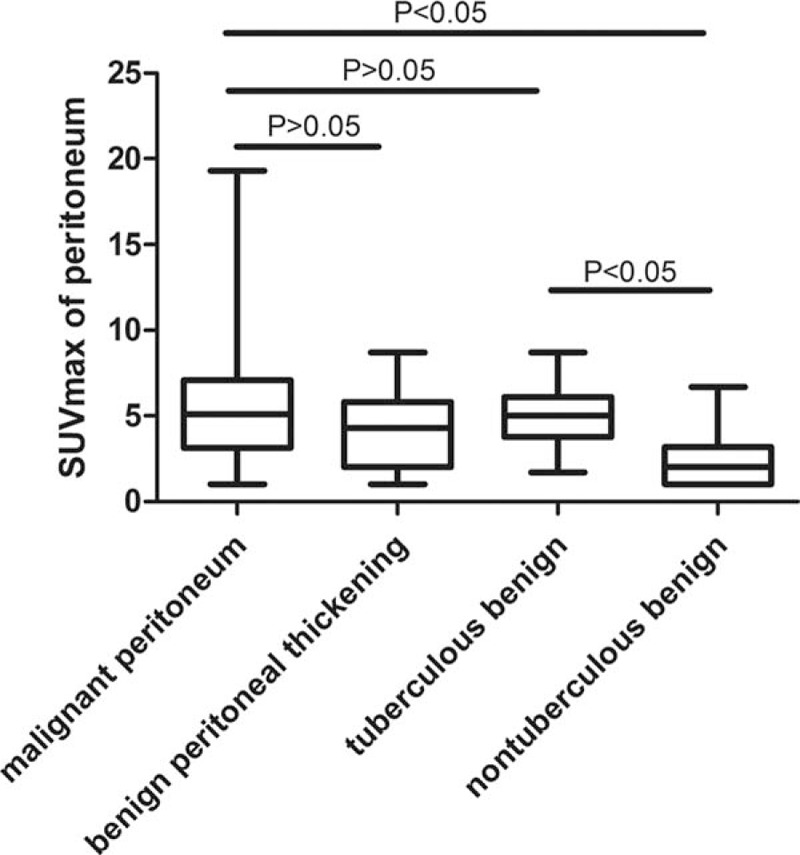
Comparison of maximal standardized uptake value (SUV_max_) of the peritoneum in malignant peritoneal thickening, benign peritoneal thickening, tuberculous peritoneal thickening, and nontuberculous peritoneal thickening (box plot graphy).

**FIGURE 5:**
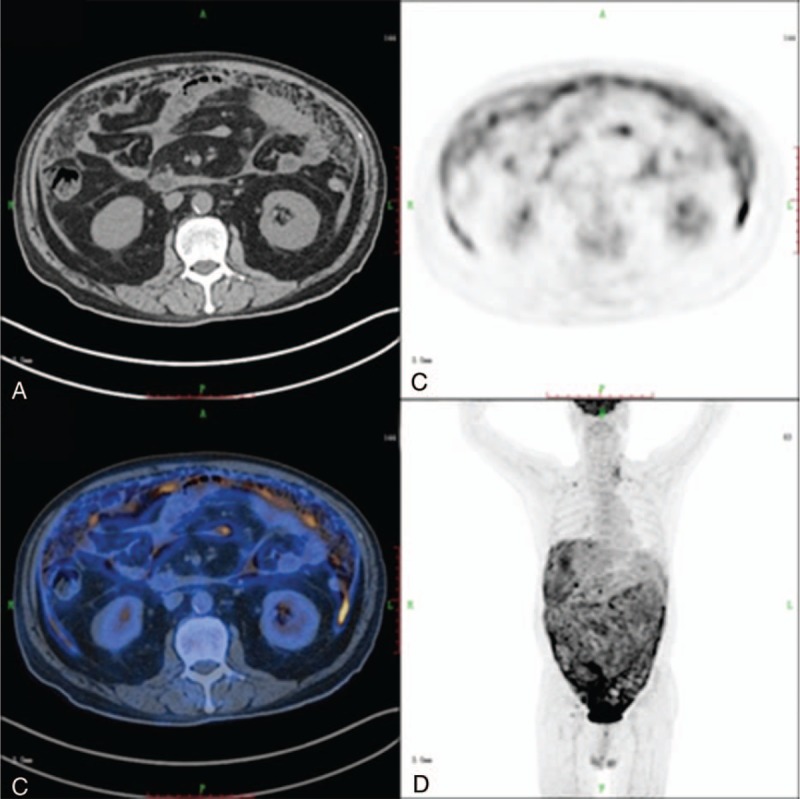
Images of 73-year-old man who went in for check-up: axial CT (A), axial PET (B), axial fused PET/CT (C), and 3D PET (D). The patient had serum CA12-5 of 246 U/mL, normal carcinoembryonic antigen, and normal CA19-9. PET/CT images show high uptake in the peritoneum (SUV_max_ of 4.6) which suggest a malignant lesion. After PET/CT examination, peritoneal biopsy confirmed that the patient had malignant peritoneal mesothelioma. CT = computed tomography, PET = positron emission tomography, SUV_max_ = maximal standardized uptake value.

**FIGURE 6:**
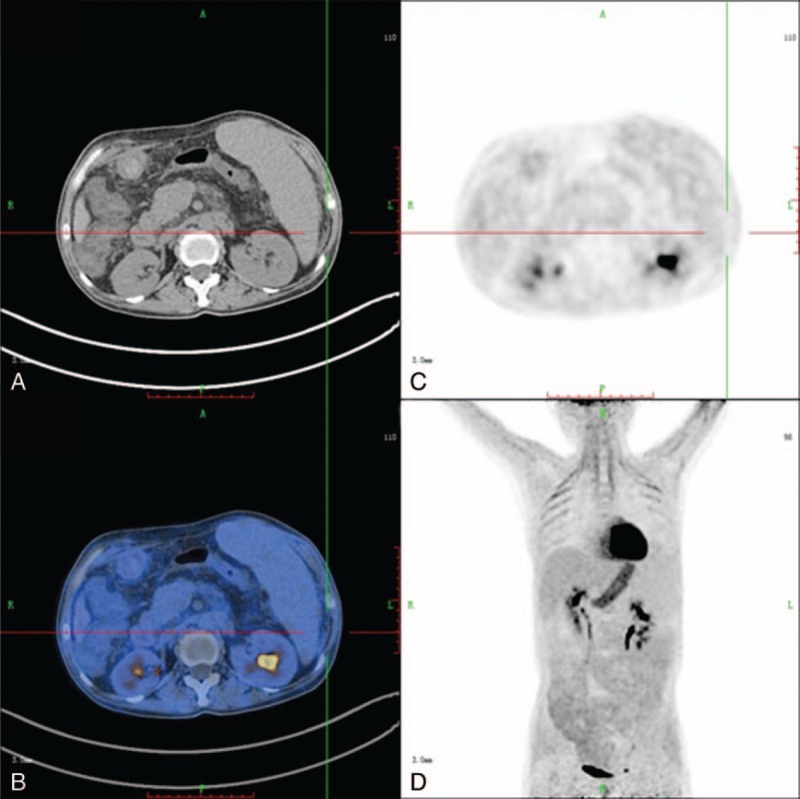
Images of 42-year-old woman who presented with fever for 1 week: axial CT (A), axial PET (B), axial fused PET/CT (C), and 3D PET (D). The patient had history of hepatic sclerosis. PET/CT images show peritoneal thickening and ascites. PET/CT images show normal uptake in the peritoneum. CT = computed tomography, PET = positron emission tomography.

Although there was no significant difference in the SUV_max_ between malignant peritoneal thickening and benign peritoneal thickening (5.407 ± 3.174 vs 4.189 ± 2.378; *P* = 0.12), the SUV_max_ was significantly higher in malignant peritoneal thickening than in nontuberculous benign peritoneal thickening (5.407 ± 3.174 vs 2.600 ± 2.036; *P* = 0.02; Figures 4–6). Among the 19 cases of benign peritoneal thickening, the SUV_max_ of the thickened peritoneum was significantly higher in tuberculous peritoneal thickening than in nontuberculous benign peritoneal thickening (5.117 ± 2.110 vs 2.600 ± 2.036; *P* = 0.02; Figure 2 and Figures 4–6). Tuberculous peritoneal thickening showed a hypermetabolic pattern, with SUV_max_ in the range of 1.7 to 8.6. Thus, there was no significant difference between malignant peritoneal thickening and tuberculous peritoneal thickening in SUV_max_, and benign tuberculous peritoneal thickening could mimic malignant peritoneal thickening on ^18^F FDG PET/CT.

To determine whether PET/CT can help differentiate between malignant and tuberculous peritoneal thickening, we further compared the differences between these 2 peritoneal thickening in PET/CT parameters and clinical characteristics (Table 2). The following significant differences were found (expressed as malignant vs tuberculous peritoneal thickening): age (61.2 ± 13.3 vs 47.6 ± 19.0 years; *P* = 0.002), pattern of peritoneal thickening (smooth 23.8% vs 66.7%; *P* = 0.005), and presence of ascites (85.7% vs 58.3%; *P* = 0.02). The other PET/CT findings and clinical characteristics evaluated, including sex, SUV_max_ of peritoneum, presence of primary lesions, and SUV_max_ of the primary lesion, were not good discriminators (*P* > 0.05 in all cases).

**TABLE 2:**
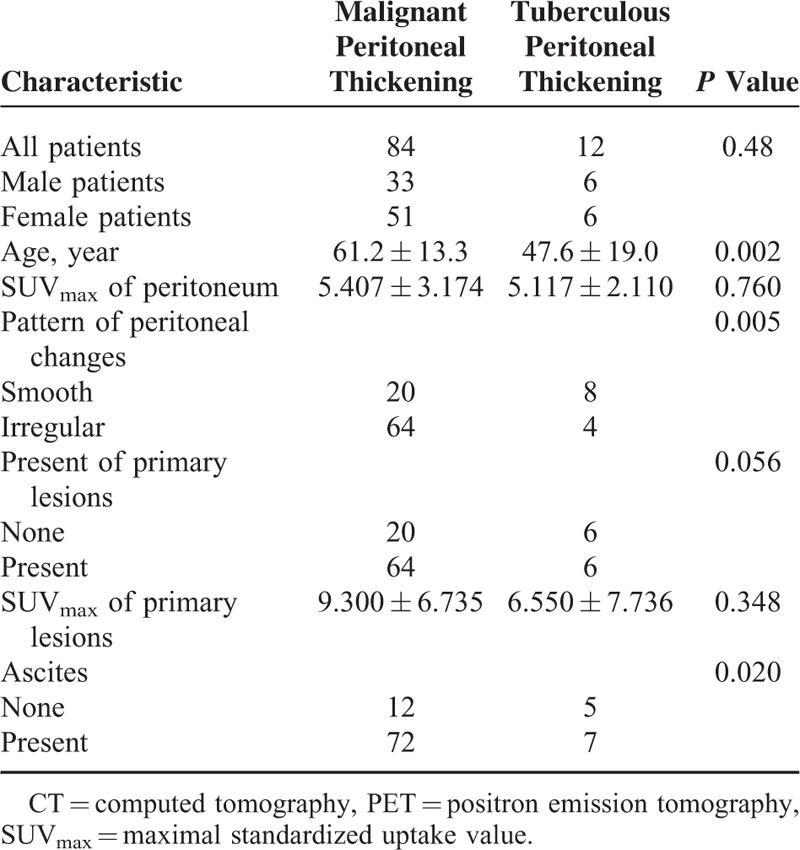
PET/CT Parameters and Association with Malignant and Tuberculous Peritoneal Thickening

## DISCUSSION

In patients with peritoneal thickening, the decision to perform surgery or undertake other treatments is ultimately made on the basis of multiple factors, including the patient's symptoms, the physical examination findings, and laboratory test results. PET/CT findings that suggest either benign or malignant causes of peritoneal thickening can help guide treatment in these patients. In this study, the most common cause of malignant peritoneal thickening was gastric cancer, followed by ovarian cancer and pancreatic cancer. The most common cause of benign peritoneal thickening was peritoneal tuberculosis.

Peritoneal thickening is known to be caused by various diseases from the entire body. Early diagnosis of the primary lesion is crucial for making an effective treatment plan and predicting prognosis. Peritoneal biopsy may have a relatively high diagnostic accuracy, but its utility is limited because of its invasiveness.^5^ PET/CT can be used to detect abnormal uptake of the whole body.^14,15^ Thus, PET/CT offers the advantage of locating both the primary lesions and peritoneal thickening. However, few studies have discussed the role of PET/CT in detecting the primary cause of peritoneal thickening, and most have focused upon its use for detecting peritoneal carcinoma.^16,17^ Therefore, we first assessed the value of PET/CT in locating the primary lesion in patients with peritoneal thickening. Our study demonstrated that the sensitivity and accuracy of PET/CT for detecting the primary lesions were superior to those of conventional CT imaging. The addition of PET/CT can reveal more information about the primary lesion and aid selection of therapeutic strategies. Our findings suggest that PET/CT should be performed in all patients with peritoneal thickening of undetermined origin so that the appropriate treatment can be determined.

However, false-positive or false-negative results are possible with PET/CT. In our study, false-positive findings were found in 4 patients (4.7%) with peritoneal tuberculosis. Previous reports show that ^18^F-FDG could accumulate at sites of inflammation and granulomatous disease,^18,19^ and our study findings were consistent with these reports. In these false-positive cases, increased ^18^F-FDG uptake may be caused by overexpression of glucose transporter isotypes and glycolytic enzymes in inflammatory cells.

Now that there were false-positive findings and benign peritoneal thickening may mimic malignant peritoneal thickening, we tried to determine whether PET/CT could help differentiate between benign and malignant peritoneal thickening. Our results showed that malignant peritoneal thickening had significantly higher SUV_max_ than nontuberculous benign peritoneal thickening; however, tuberculous peritoneal thickening also had high SUV_max_ and could thus mimic malignant peritoneal thickening. Previous studies have also reported that tuberculous peritonitis shows a hypermetabolic pattern that might mimic peritoneal carcinoma.^20–22^ Tuberculosis is composed of lymphocytes and macrophages. These inflammatory cells have markedly increased glycolysis, which is the cause of increased ^18^F-FDG uptake.^22^ However, there were multiple PET/CT parameters that were significantly different in patients with tuberculous compared with those with malignant causes of peritoneal thickening in our study. These included age, pattern of peritoneal thickening, and presence of ascites. Patients with malignant peritoneal thickening were older than patients with tuberculous peritoneal thickening; they were also more likely to have ascites and an irregular pattern of peritoneal changes. So, when we make the differential diagnosis between malignant and tuberculous peritoneal thickening, these PET/CT parameters should be taken into consideration.

To the best of our knowledge, this is the 1st large study on the ability of ^18^F-FDG PET/CT to characterize peritoneal thickening of undetermined origin. This study has some limitations. First, the study was retrospective and there was unavoidable selection bias. Second, we had a small and heterogeneous sample, with patients having a variety of different malignancies and therapies.

Based on our results the use of PET/CT led to high sensitivity and specificity in diagnosing peritoneal thickening of undetermined origin. Malignant peritoneal thickening had a significantly higher SUV_max_ than nontuberculous benign peritoneal thickening. However, tuberculous peritoneal thickening also has a high SUV_max_, which may mimic malignant peritoneal thickening. Multiple factors that were found to be different between patients with tuberculous and malignant peritoneal thickening should be taken into consideration when making the differential diagnosis. The change in SUV_max_ or metabolic tumor volume in the follow-up scans can be mentioned in future studies which may comment upon sensitivity and other parameters. Further large prospective studies, with histological examination as the reference standard, are needed to confirm our results and determine whether they can be applied to healthy patients with incidentally discovered peritoneal thickening.
